# Editorial: Targeting Monocytes/Macrophages to Treat Atherosclerotic Inflammation

**DOI:** 10.3389/fphar.2020.00086

**Published:** 2020-02-14

**Authors:** Alessandro Corti, Caroline Gaucher, Alfonso Pompella

**Affiliations:** ^1^Dipartimento di Ricerca Traslazionale NTMC, Scuola Medica dell'Università di Pisa, Pisa, Italy; ^2^Université de Lorraine, CITHEFOR, Nancy, France

**Keywords:** monocytes/macrophages, inflammation, foam cell formation, smooth muscle cells, atherosclerosis progression

By now well into the XXI century, atherosclerosis with its cerebro- and cardiovascular complications continue to represent the leading cause of morbidity and mortality at global level. The identification and control of known risk factors—hypertension, diabetes, cigarette smoking and above all, elevated low-density lipoprotein (LDL) cholesterol—have actually allowed the achievement of a dramatic decrease in the incidence of major cardiovascular diseases in many western countries, and current pharmacological treatments aiming to slow down progression of atherosclerosis are thus almost entirely centered on reducing plasma cholesterol levels. Nevertheless, elevated LDL levels alone cannot account for the entire burden of atherosclerosis, and the concept of atherosclerosis as a *proliferative* disorder independent of cholesterol levels has gained increased significance as a pathogenic pathway since its first proposal back in the ‘70s (Ross and Glomset, 1973). It is by now widely acknowledged that the pathogenic basis of atherosclerosis extends far beyond intimal infiltration of cholesterol. Animal experiments, observations on human atheromata as well as clinical biomarker studies, all support the importance of immune and inflammatory pathways in the initiation, progression, and eventual thrombotic manifestations of the disease.

The precise identification of the cell types accumulating inside human atherosclerotic lesions had to wait until the advent of monoclonal antibody technology, which eventually allowed to recognize mononuclear phagocytes as the main precursors of “foam cells” typically populating the plaques. The same was then observed for smooth muscle cells of the arterial wall, which can also originate macrophage-like and even typical foam cells through sort of a metaplastic transformation (Bennett et al., 2016). The “proliferative” and the “inflammatory” interpretations of atherogenesis can be thus reunified into one comprehensive theory, viewing atherosclerosis as an extraordinarily complex pathobiological process in which pathways of inflammation are set into motion by several risk factors and in turn promote altered behaviors of arterial wall cells. In this picture, cytokines, chemokines, and adhesion molecules associated with components of the vessel wall, as well as the immune/inflammatory cell types intervening in the lesions—monocytes/macrophages in the first place—become the natural subjects for investigation, being the likely responsible of altered arterial biology. Among these, several potential targets for therapeutic treatments of atherosclerosis have been conjectured. Indeed, the translation of biological insights into new solutions is starting to work, as shown by the encouraging results of antiinflammatory treatments based on anti-IL-1beta monoclonal antibodies [CANTOS trial: (Libby, 2017; Ridker et al., 2017)].

The reviews and research articles that make up the present Research Topic represent a unique collection, capable of providing an overview of current trends of pharmacological research in the atherosclerosis field. To start with, the paper by Flynn et al. provides an overview of the different types and origin of macrophages and macrophage-like cells contributing to the atherosclerotic disease. The focus is mainly pointed on the influence of diabetes and obesity on myelopoiesis and macrophage activation/accumulation, leading to an overall increased cardiovascular risk. Targeting the production of monocyte-derived macrophages was shown to reduce preclinical atherosclerosis in a number of metabolic and inflammatory diseases. A key role is played by S100A8/A9 heterocomplex, a myeloproliferative factor active both in diabetes and obesity, whose inhibition could represent a strategy to reduce cardiovascular risk in this kind of patients. The following paper by Martinet et al. proposes an interesting insight into the macrophage death modes observed in advanced plaques, from canonical (necrosis, apoptosis) to more exotic ones (efferocytosis, necroptosis, pyroptosis, ferroptosis, parthanatos, as well as autophagic death).

A correct appraisal of these processes is obviously crucial for the development of pharmacological interventions aiming at stabilization of vulnerable, rupture-prone plaques, or possibly even at regression of lesions. The remaining papers in the present series in fact are all examples of this kind of approach. Nikiforov et al. provide, *e.g.*, an overview of studies on the activation status of monocytes in atherosclerotic patients. Monocytes obtained from atherosclerotic patients indeed present with a hyperreactive, “trained” phenotype which significantly correlates with intima-media thickness taken as an index of disease progression. The authors propose thus to employ trained monocytes from atherosclerotic patients as an *ex vivo* model for testing the ability of potential antiatherogenic compounds to attenuate the monocyte hyperreactivity.

Can any clues be derived from epigenetics? In their elegant original article, Luque-Martin et al. have investigated the antiinflammatory effects of an esterase-sensitive histone deacetylase inhibitor. The authors do observe a reduced production of proinflammatory cytokines by isolated peritoneal macrophages. On the other hand results in an *in vivo* knock-out (ldlr-/-) mice model were disappointing, as the inhibitor could not reduce the formation of plaques. Nevertheless, the study overall offers a remarkable example of how efficient experimental strategies can be devised.

The review by Getz and Reardon explores the structure-function relationships of apoproteins (apoE, apoA-I) and serum amyloid A (SAA) with their ability to regulate cholesterol homeostasis within macrophages. Mimetic peptides derived from the three apoproteins are proposed as therapeutic agents. ApoE- as well as SAA-mimetic peptides were shown, *e.g.*, to reduce atherosclerosis in apoE^-/-^ mice. ApoA-1-mimetic is probably the most promising compound, since its antiinflammatory potential has already been shown in other inflammatory disorders such as respiratory and intestinal diseases and chronic arthritis.

Vascular smooth muscle cells (VSMCs) are the subject of the review by Ramel et al. Plasticity of VSMCs during the progression of atherosclerosis, as well as their complex interactions with endothelium and monocytes/macrophages are comprehensively overviewed. A general but detailed picture is thus provided of what the authors call *“a bad dialogue”* taking place between VSMCs and immune cells, capable of modulating plaque stability vs. progression and rupture. Against this background, experimental studies investigating a series of potential molecular targets for therapeutic intervention—IL-1beta, histone H4, chemokine CXCL10, etc.—are reviewed. The paper by Pastore et al. focuses instead on the myeloid-epithelial-reproductive tyrosine kinase (MerTK), a factor involved in shaping macrophages differentiation towards a M2, “reparative” phenotype. The role of MerTK in nonalcoholic fatty liver disease (NAFLD)-associated cardiovascular diseases is highlighted, together with its possible use as an innovative target. Interestingly, both a selective PPAR-γ antagonist and a synthetic agonist for liver “X” receptors (LXRs) can upregulate MerTK expression. The authors overview the small-molecule MerTK inhibitors and monoclonal antibodies currently under evaluation.

The importance of miRNAs in the development and progression of atherosclerosis is receiving increasing attention. The mini-review by Bruen et al. deals with the inhibition of macrophage-specific micro-RNA miR-155 as a viable therapeutic strategy to decrease inflammation. Indeed, conjugated linoleic acid (CLA) as well as PPAR-γ agonists were shown to regulate candidate miRNAs and promote a proresolving atherosclerotic plaque microenvironment. At present however no miRNA-based anti-atherosclerotic therapies have yet entered clinical trials, since the specific delivery of miRNAs to the desired sites of action—which is mandatory in order to prevent off-target effects—is still not feasible.

Last but not least, the contribution by van der Vorst et al. is an appraisal of the G-protein coupled receptors (GPCRs) in the inflammatory process. A selection of GPCRs mainly expressed on myeloid cells are discussed as potential players in progression of atherosclerosis. In particular, GPCRs working as receptors for chemokines and formyl-peptide, chemerin receptor 23, as well as the calcium-sensing receptor are taken into account as potential targets for treatment of cardiovascular diseases.

In conclusion, nobody can tell how long we will have to wait before adequate anti-atherosclerotic treatments become available in the clinic. The complexity of the matter has even led someone to question whether atherosclerosis truly represents a single pathological condition, or rather the term actually comprises many disease “subtypes” (Khera and Kathiresan, 2017). Difficult obstacles in the way of research remain to be overcome. As shown however by the recent literature—including the present Research Topic—the investigative efforts in search of possible leads to therapy are gradually yielding some first translational, intriguing results.

## Author Contributions

AC, CG, and AP discussed the contents of the paper. AP drafted the manuscript.

## Conflict of Interest

The authors declare that the research was conducted in the absence of any commercial or financial relationships that could be construed as a potential conflict of interest.
